# Dataset on effect of decolourisation on metabolomic profile of *Moringa oleifera* leaf powder

**DOI:** 10.1016/j.dib.2022.108508

**Published:** 2022-08-04

**Authors:** Adewumi Toyin Oyeyinka, Oluwafemi Ayodeji Adebo, Muthulisi Siwela, Kirthee Pillay

**Affiliations:** a Discipline of Dietetics and Human Nutrition, School of Agricultural, Earth and Environmental Sciences, University of KwaZulu-Natal, South Africa; b Biotechnology and Food Technology, University of Johannesburg, South Africa

**Keywords:** Metabolites, GC-HRTOF-MS, Moringa decolourisation, Fortification

## Abstract

Moringa leaf has been widely used in the enrichment of staple foods due to its high nutritional value and hypoglycaemic, immune boosting, antiviral, antioxidant and antimicrobial activities. However, the acceptability of these products is generally low due to the green colour imparted by the colour of Moringa leaf. Decolourisation of the leaves may improve the acceptability of the food products. The decolorisation process may not only change the chlorophyll concentration of the Moringa leaves but also its other chemical components. The data set describes the effect of decolourisation on the metabolites present in Moringa leaf powder. The raw and decolourised samples were extracted with methanol/water (80:20 v/v) and analysed using a gas chromatography-high resolution time of flight-mass spectrometer (GC-HRTOF-MS). The metabolites identified were classified based on their functional group into acids, alcohols, aldehydes, amides hydrocarbons, phenols, phytosterols, vitamins and others. The data presented can be useful in identifying functional compounds available in Moringa-based foods and understanding the effect of decolourisation on the metabolite profile.

## Specifications Table

**Table:** 

Subject	Food Science: Food Chemistry
Specific subject area	Processing; Pigment extraction; Metabolomics
Type of data	Table; Figure; Spectra data
How the data were acquired	*Moringa oleifera* leaf powder (MOLP) was decolourised by homogenising the leaf powder with ethanol (95%) at a powder to solvent ratio of 1:20, placed on an orbital shaker at 150 rpm for 30 min. The samples were later centrifuged, supernatant discarded, and the slurry dried at 40 °C. Metabolites in the raw and decolourised powder were extracted with methanol/water at 80:20 v/v. The extracts were analysed using the LECO Pegasus GC-HRTOF-MS system (LECO Corporation, St Joseph, USA) fitted with resolution of 50,000 FWMH (full peak with at one half maximum), with mass accuracies/errors of < 1 ppm and acquisition rates of up to 200 spectra/s. The system is equipped with an Agilent 7890A gas chromatograph (Agilent Technologies, Inc., Wilmington, DE, USA), Gerstel MPS multipurpose autosampler (Gerstel Inc., Mülheim an der Ruhr, Germany) and a Rxi ®-5ms column (30 m × 0.25 mm ID × 0.25 μm) (Restek, Bellefonte, USA).
Data format	Raw and analysed data
Description of data collection	A single biological Moringa leaf powder sample was used in the experiment. The sample was extracted in duplicate, and the duplicate extracts injected in triplicate. Raw and decolourised leaf powder (1 g) was extracted with 10 ml extraction solvent (methanol/water 80:20 v/v). The extract was concentrated, reconstituted in 1 ml methanol (99.9% pure chromatography grade) and filtered into dark vials using 0.22 μm syringe filters. Afterwards, 1 µl of sample was auto-injected to the GC-HRTOF-MS machine and metabolite identities were determined using NIST, Mainlib and Feihn metabolomics databases.
Data source location	MOLP was sourced from the Agricultural Research Council (ARC), Pretoria, Gauteng, South Africa (S 25° 44′ 55. 8″ E 28° 14′ 14. 0″) and analyses were done at the University of Johannesburg (Doornfontein Campus), Johannesburg, South Africa (S 26° 11′ 32. 6″ E 28° 03′ 28. 9″).
Data accessibility	Raw and processed dataset have been deposited in Mendeley repository and is accessible using the link: DOI: 10.17632/7mrhxrt9kr.1; https://data.mendeley.com/datasets/7mrhxrt9kr/1[1]

## Value of the Data

• The data present the effect of decolourisation on the metabolite profile of Moringa leaf powder samples and give an insight to metabolite modifications after decolourisation.
• The data reported would be useful to food processors for supplementing staple foods with decolourised moringa leaf powder for improved nutrition.
• The data could be useful for comparative analysis of metabolite composition of moringa leaf powder grown in different locations and may be useful for selecting moringa leaf species for various food and non-food applications.

## 1 Data Description

**Fig. 1:**
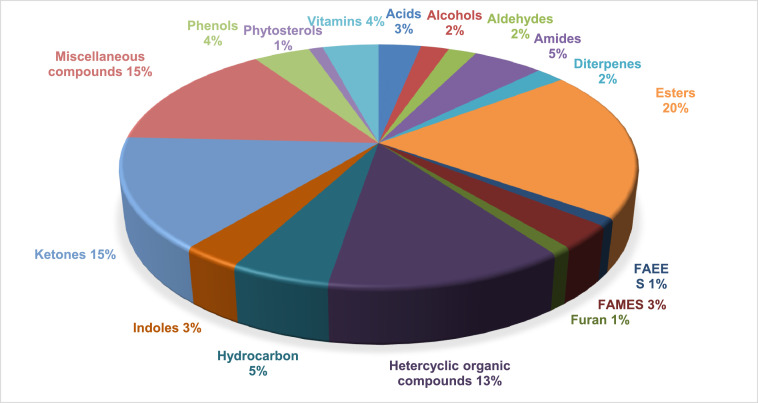
Pie chart indicating the percentage distribution of the compounds in the raw Moringa leaf powder.

**Fig. 2:**
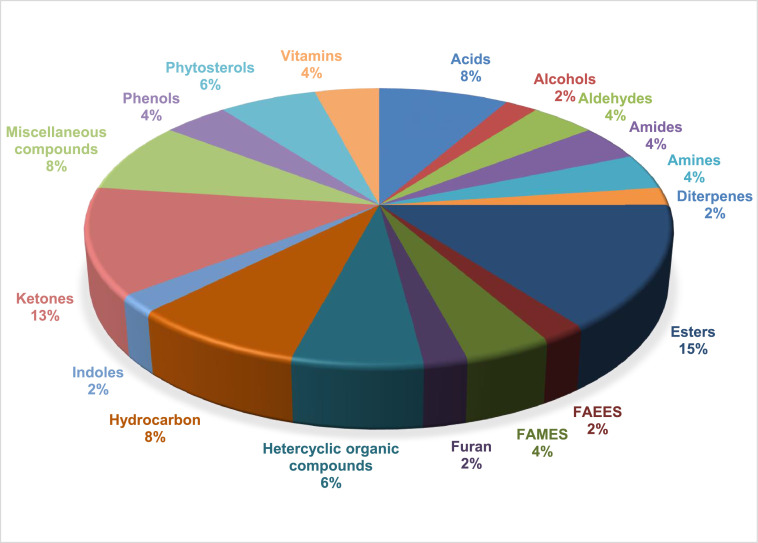
Pie chart indicating the percentage distribution of the compounds in the decolourised Moringa leaf powder.

The metabolite data obtained from raw and decolourised MOLP are presented. Table 1 represents the metabolites obtained from MOLP. The data in the table includes the following: retention time, observed mass, metabolite name, molecular formula and average peak area for each metabolite identified in the different samples. These were obtained from the peaks generated from GC-HRTOF-MS analysis and comparison of spectra obtained with NIST, Mainlib and Feihn metabolite databases. The raw and analysed data together with the spectra obtained are available as supplementary documents (https://data.mendeley.com/datasets/7mrhxrt9kr/1) [1]. Figs. 1 and 2 summarises the percentage distribution of the compound groups in the raw and decolourised samples, respectively.

**Table 1:** Metabolites Identified in Raw and Decolourised Moringa Leaf Powder.

				Average Peak Area	
RT (Min)	Observed Ion m/z	Name	Molecular Formula	Raw	Decolourised
Acids					

9:33	150.0675	Hydrocinnamic acid	C_9_H_10_O_2_	267444 ± 1677	ND
15:90	207.1833	Tetradecanoic acid^a^	C_14_H_28_O_2_	870892 ± 1728	112615 ± 1544
15:85	171.1378	n-Decanoic acid	C_10_H_20_O_2_	ND	136420 ± 955
18:31	256.2399	n-Hexadecanoic acid^a^	C_16_H_32_O_2_	20230631 ± 3875	4957168 ± 1078
20:20	178.1343	9,12,15-Octadecatrienoic acid, (Z,Z,Z)-	C_18_H_30_O_2_	ND	385144 ± 848

Alcohol					

3:50	87.0439	2-Chloroethanol	C_2_H_5_ClO	ND	6540954 ± 381
3:10	98.0361	2-Furanmethanol	C_5_H_6_O_2_	2332645 ± 2154	ND
4:24	110.036	(3-Fluorophenyl) methanol, n-butyl ether	C_11_H_15_FO	2215455 ± 1928	ND

Aldehyde					

4:23	110.0361	2-Furancarboxaldehyde, 5-methyl-	C_6_H_6_O_2_	4329276 ± 1750	ND
5:29	120.0568	Benzeneacetaldehyde	C_8_H_8_O	5401177 ± 2096	ND
7:56	120.0569	Benzaldehyde, 3-methyl-	C_8_H_8_O	ND	310003 ± 0
7:66	120.0570	Benzaldehyde, 2-methyl-	C_8_H_8_O	709831 ± 691	ND
20:00	264.2451	9,12,15-Octadecatrienal	C_18_H_30_O	ND	3090025 ± 600

Amide					

11:23	174.0667	Furmecyclox	C_14_H_21_NO_3_	90492 ± 156	ND
12:23	193.1693	Benzamide, 2,6-difluoro-N-heptyl-	C_14_H_19_F_2_NO	548309 ± 1639	ND
14:65	197.1204	Formamide, N-(1,1′-biphenyl)-2-yl-	C_13_H_11_NO	49904 ± 461	ND
19:65	193.0890	1-Anthracenamine	C_14_H_11_N	63892 ± 187	ND
19:93	154.1226	Dodecanamide, N-(2-hydroxyethyl)-	C_14_H_29_NO_2_	ND	82268 ± 779
25:16	172.1561	9-Octadecenamide, (Z)- b	C_18_H_35_NO	297218 ± 1354	212162 ± 1040

Amines					

12:99	143.0730	2-Naphthalenamine	C_10_H_9_N	ND	27586 ± 1359
20:07	243.2511	Ethanol, 2,2′-(dodecylimino)bis-	C_16_H_35_NO_2_	ND	74343 ± 575

Diterpenes					

17:30	207.7144	Neophytadiene^a^	C_20_H_38_	383948 ± 846	115003 ± 564
19:70	279.3006	Phytol	C_20_H_40_O	2504053 ± 1783	ND
Esters					

5:97	134.0835	1,2-Ethanediol, dipropanoate	C_8_H_14_O_4_	ND	1085862 ± 1179
6:01	112.0394	dl-Alanine ethyl ester	C_5_H_11_NO_2_	5911350 ± 2645	ND
6:55	168.9881	Glycine, N-methyl-N-methoxycarbonyl-, undecyl ester	C_16_H_31_NO_4_	ND	435634 ± 690
7:41	149.1073	Terephthalic acid, 4-fluorophenethyl octyl ester	C_24_H_29_FO_4_	104029 ± 1101	ND
11:23	133.1011	4-Fluorobenzoic acid, tridec-2-ynyl ester	C_20_H_27_FO_2_	87384 ± 368	ND
11:55	176.0829	3-Butenoic acid, 4-phenyl-, methyl ester^a^	C_11_H_12_O_2_	245772 ± 1255	91379 ± 433
11:96	166.0624	Benzeneacetic acid, 4-hydroxy-, methyl ester	C_9_H_10_O_3_	ND	183725 ± 496
12:23	161.5899	2,4-Difluorobenzoic acid, 2-formyl-4,6-dichlorophenyl ester^a^	C_14_H_6_C_l2_F_2_O_3_	442533 ± 587	106647 ± 943
12:41	178.0623	4-Fluorobenzoic acid, tridec-2-ynyl ester	C_20_H_27_FO_2_	64013 ± 325	ND
13:32	168.0414	Fumaric acid, ethyl 3,4,5-trichlorophenyl ester	C_12_H_9_Cl_3_O_4_	ND	74561 ± 613
13:39	139.0377	Fumaric acid, ethyl 2,3,5-trichlorophenyl ester	C_12_H_9_Cl_3_O_4_	220060 ± 963	ND
17:19	150.0264	Phthalic acid, monoamide, N-ethyl-N-(3-methylphenyl)-, isobutyl ester	C_21_H_25_NO_3_	ND	49831 ± 575
17:21	224.1004	1,2-Benzenedicarboxylic acid, bis(2-methylpropyl) ester	C_16_H_22_O_4_	209387 ± 1458	ND
18:22	223.0960	Dibutyl phthalate	C_16_H_22_O_4_	237942 ± 909	ND
21:23	157.0838	Carbonic acid, 2-dimethylaminoethyl isobutyl ester	C_9_H_19_NO_3_	225107 ± 1068	ND
21:24	179.0012	Octanoic acid, 2-dimethylaminoethyl ester	C_12_H_25_NO_2_	173798 ± 867	ND
22:74	88.0757	Cyclobutanecarboxylic acid, 2-dimethylaminoethyl ester	C_9_H_17_NO_2_	345374 ± 1472	ND
23:11	300.2600	Hexadecanoic acid, 2-hydroxy-1-(hydroxymethyl)ethyl ester	C_19_H_38_O_4_	1227632 ± 1574	ND
24:67	285.2783	Pentadecanoic acid, 2-hydroxy-1-(hydroxymethyl)ethyl ester	C_18_H_36_O_4_	306505 ± 799	ND
24:67	285.2781	Glycerol 1-palmitate	C_19_H_38_O_4_	414699 ± 1891	ND
24:67	285.2778	Pentadecanoic acid, 2-hydroxy-1-(hydroxymethyl)ethyl ester	C_18_H_36_O_4_	393653 ± 1489	ND
27:15	210.0300	2-Amino-3-cyano-4-methyl-4,6-bis-(5-nitro-benzofuran-2-yl)-cyclohexa-1,5-dien-1,3-dicarboxylic acid, diethyl ester	C_30_H_24_N_4_O_10_	39484 ±128	ND
27:16	204.1105	4′-Cyano-4-biphenylyl p-heptylbenzoate	C_27_H_27_NO_2_	121833 ± 985	ND
27:16	414.3457	2-Amino-3-cyano-4-methyl-4,6-bis-(5-nitro-benzofuran-2-yl)-cyclohexa-1,5-dien-1,3-dicarboxylic acid, diethyl ester	C_30_H_24_N_4_O_10_	137111 ± 568	ND

Fatty Acid Ethyl Esters					

18:46	241.2156	Pentadecanoic acid, ethyl ester	C_17_H_34_O_2_	ND	142849 ± 872
23:12	300.2615	Hexadecanoic acid, 2-hydroxy-1-(hydroxymethyl)ethyl ester	C_19_H_38_O_4_	652035 ± 1645	ND
Fatty Acid Methyl Esters					

17:75	270.2551	Pentadecanoic acid, 14-methyl-, methyl ester	C_17_H_34_O_2_	ND	452607 ±1184
17:76	228.2046	Tridecanoic acid, methyl ester	C_14_H_28_O_2_	577213 ± 1215	ND
17:76	213.1850	Dodecanoic acid, methyl ester	C_13_H_26_O_2_	690741 ± 783	ND
19:57	236.1769	9,12,15-Octadecatrienoic acid, methyl ester, (Z,Z,Z)-	C_19_H_32_O_2_	523501 ± 964	ND
19:77	255.2315	Tridecanoic acid, methyl ester	C_14_H_28_O_2_	ND	118000 ± 674

Furan					

7:91	126.0311	5-Hydroxymethylfurfural^a^	C_6_H_6_O_3_	12643146 ± 4287	4540831 ± 651

Heterocyclic organic compounds					

3:90	85.0884	2H-Pyran-2,3-diol, tetrahydro-, diacetate, trans-	C_9_H_14_O_5_	ND	717213 ± 986
5:73	136.0995	Pyrazine, 2,6-diethyl-	C_8_H_12_N_2_	258433 ± 1782	ND
6:56	117.0572	Benzene, 1-isocyano-2-methyl-	C_8_H_7_N	96307 ±136	ND
6:61	134.0837	5H-5-Methyl-6,7-dihydrocyclopentapyrazine	C_8_H_10_N_2_	66695 ± 941	ND
8:09	164.1433	2-Isoamyl-6-methylpyrazine	C_10_H_16_N_2_	271657 ± 1857	ND
8:09	150.0916	2-Butyl-3-methylpyrazine	C_9_H_14_N_2_	346420 ±1264	ND
8:15	142.0624	3-Oxo-4-phenylbutyronitrile	C_10_H_9_NO	861303 ± 987	ND
8:97	163.1227	2-(3-Methylbutyl)-3,5-dimethylpyrazine	C_11_H_18_N_2_	236451 ± 1057	ND
99:00	150.0676	Pyrazine, 3,5-dimethyl-2-propyl-	C_9_H_14_N_2_	ND	3873 ± 58
10:10	132.0682	N-Ethyl-2-benzyloxycarbonylazetidine	C_13_H_17_NO_2_	128658 ± 865	ND
11:11	176.0831	Methyl trans-2-phenyl-1-cyclopropanecarboxylate	C_11_H_12_O_2_	57884 ± 212	ND
11:17	156.1017	3-Nitrobenzyl iodide	C_7_H_6_INO_2_	ND	117173 ± 381
13:26	183.0912	2H-1-Benzopyran-3,4-diol, 2-(3,4-dimethoxyphenyl)-3,4-dihydro-6-methyl-, (2a,3a,4a)-	C_18_H_20_O_5_	57984 ± 126	ND
14:62	182.0945	Benzenepropanol, 4-hydroxy-3-methoxy-	C_10_H_14_O_3_	282101 ± 362	ND
18:48	239.2370	a-D-Glucopyranoside, methyl 2,3,4-tri-O-methyl-	C_10_H_20_O_6_	134947 ± 783	ND

Hydrocarbons					

5:59	95.0332	Dipivefrine, N.O-bis(pentafluoropropionyl)-	C_25_H_27_F_10_NO_7_	4887767 ± 1892	ND
5:82	120.0566	1,3,5,7-Tetroxane	C_4_H_8_O_4_	28996026 ± 3624	ND
10:30	126.0467	2,2-Dichloroethyl methyl ether	C_3_H_6_Cl_2_O	3102554 ± 1560	ND
10:61	172.1245	(E)-1-(2,3,6-trimethylphenyl)buta-1,3-diene (TPB, 1)	C_13_H_16_	57373 ±657	ND
11:03	133.0918	5-Trimethylsilylpent-2-en-4-yne^a^	C_8_H_14_Si	51869 ± 235	30748 ± 464
12:03	326.9667	3-Butoxy-1,1,1,7,7,7-hexamethyl-3,5,5-tris(trimethylsiloxy)tetrasiloxane	C_19_H_54_O_7_Si_7_	ND	60326 ± 837
14:64	401.9860	Cyclooctasiloxane, hexadecamethyl-	C_16_H_48_O_8_Si_8_	ND	107780 ± 553
20:20	173.1325	9,12,15-Octadecatrien-1-ol, (Z,Z,Z)-	C_18_H_32_O	ND	285352 ± 848

Indole					

8:73	117.0571	Indole^a^	C_8_H_7_N	187094 ± 961	81834 ± 818
19:62	193.0884	3-Phenylindole	C_14_H_11_N	26717 ± 561	ND
19:68	278.2972	Phytol	C_20_H_40_O	1042771 ± 964	ND

Ketones					

3:38	96.0205	4-Cyclopentene-1,3-dione	C_5_H_4_O_2_	472244 ± 425	ND
4:39	144.0415	2,4-Dihydroxy-2,5-dimethyl-3(2H)-furan-3-one^a^	C_6_H_8_O_4_	5669648 ± 3879	722966 ± 1181
5:54	128.0468	Furaneol	C_6_H_8_O_3_	14722529 ± 6465	ND
6:81	144.0418	4H-Pyran-4-one, 2,3-dihydro-3,5-dihydroxy-6-methyl-a	C_6_H_8_O_4_	407796894897	11673960 ± 3650
7:58	120.0569	Benzofuran, 2,3-dihydro-	C_8_H_8_O	ND	241287 ± 697
7:32	142.0261	4H-Pyran-4-one, 3,5-dihydroxy-2-methyl-a	C_6_H_6_O_4_	718472 ± 869	207912 ± 1452
8:98	150.0675	Ethanone, 1-(2-hydroxy-5-methylphenyl)-	C_9_H_10_O_2_	219210 ± 963	ND
10:43	206.1299	1-(3,6,6-Trimethyl-1,6,7,7a-tetrahydrocyclopenta[c]pyran-1-yl)ethenone	C_13_H_18_O_2_	25292 ± 563	ND
10:79	120.0679	7-Chloro-1,3,4,10-tetrahydro-10-hydroxy-1-[[2-[1-pyrrolidinyl]ethyl]imino]-3-[3-(trifluoromethyl)phenyl]-9(2H)-acridinone	C_26_H_25_ClF_3_N_3_O_2_	5330994 ± 3056	ND
11:95	190.1355	4-(2,6,6-Trimethylcyclohexa-1,3-dienyl)but-3-en-2-one	C_13_H_18_O	1027634 ± 2786	ND
12:60	162.0674	5-Hydroxy-3-methyl-1-indanone	C_10_H_10_O_2_	144778 ± 845	ND
13:65	194.0576	Butyrovanillone	C_11_H_14_O_3_	240775 ± 982	ND
14:27	190.1353	Megastigmatrienone	C_13_H_18_O	121488 ± 569	ND
15:13	193.1226	Methanone, (1-hydroxycyclohexyl)phenyl-	C_13_H_16_O_2_	118337 ± 678	ND
14:02	174.0991	4-Phenyl-3-penten-2-one p-toluenesulfonylhydrazone	C_18_H_20_N_2_O_2_S	ND	61326 ± 323
15:12	175.1116	Methanone, (1-hydroxycyclohexyl)phenyl-	C_13_H_16_O_2_	ND	85928 ± 476
29:19	379.3343	Allopregnane-3ß,7a,11a-triol-20-one	C_21_H_34_O_4_	1216244 ± 1985	ND

Miscellaneous compounds					

7:62	154.0625	4-tert-Butoxystyrene	C_12_H_16_O	753299 ± 869	ND
9:16	137.0468	N-(2-Propynyl)-2-methylpiperidine	C_9_H_15_N	33347 ± 155	ND
9:68	172.1245	Naphthalene, 1,2-dihydro-1,1,6-trimethyl-	C_13_H_16_	106374 ± 563	ND
10:21	119.0851	N-Ethyl-2-carbethoxyazetidine	C_8_H_15_NO_2_	ND	3080824 ± 1883
10:32	172.1245	1, 1, 5-Trimethyl-1, 2-dihydronaphthalene	C_13_H_16_	22190 ± 209	ND
11:35	173.0961	Acetyl eugenol	C_12_H_14_O_3_	66559 ± 865	ND
11:60	172.1245	Naphthalene, 1,2-dihydro-1,4,6-trimethyl-	C_13_H_16_	43628 ± 101	ND
11:64	155.0730	Pyridine, 3-phenyl-a	C_11_H_9_N	106819 ± 1796	39467 ± 567
12:13	133.0522	Benzeneacetonitrile, 4-hydroxy-a	C_8_H_7_NO	5692313 ± 2058	6655507 ± 2504
12:46	169.0881	Pyridine, 2-(4-methylphenyl)-	C_12_H_11_N	33241 ± 456	ND
12:49	142.0777	Piperidine, 1-butyl-	C_9_H_19_N	157108 ± 985	ND
14:04	183.1193	1,1,4,5,6-Pentamethyl-2,3-dihydro-1H-indene^a^	C_14_H_2_0	333883 ± 1798	56509 ± 306
16:46	166.0625	3-Methyl-5-nonylpyrrolizidine	C_17_H_33_N	136101 ± 826	ND
17:86	194.0771	Phosphine, cyclohexyl[2-(2-pyridyl)ethyl]-	C_13_H_20_NP	105122 ± 1419	ND
21:88	189.0758	4,8,12,16-Tetramethylheptadecan-4-olide	C_21_H_40_O_2_	108316 ± 746	ND

Phenols					

8:76	138.0674	Phenol, 4-(methoxymethyl)-	C_8_H_10_O_2_	149528 ± 1432	ND
15:80	180.0782	(E)-4-(3-Hydroxyprop-1-en-1-yl)-2-methoxyphenol^a^	C_10_H_12_O_3_	148123 ± 979	59968 ± 566
12:35	206.1664	2,4-Di-tert-butylphenol^a^	C_14_H_22_O	111246 ± 1189	91836 ± 684
12:35	206.1657	Phenol, 2,5-bis(1,1-dimethylethyl)-	C_14_H_22_O	125015 ± 1275	ND

Phytosterol					

29:05	415.3873	Cholesterol 3-O-[[2-acetoxy]ethyl]-	C_31_H_52_O_3_	ND	436680 ± 1045
29:17	315.2636	Cholesta-8,24-dien-3-ol, 4-methyl-, (3ß,4a)-	C_28_H_46_O	ND	213935 ± 1115
29:40	341.8057	ß-Amyrin^a^	C_30_H_50_O	487813 ± 1542	78184 ±465
29:07	416.3920	ß-Sitosterol acetate	C_31_H_52_O_2_	ND	ND

Vitamins					

25:75	420.3556	a-Tocospiro B	C_29_H_50_O_4_	40453 ± 1006	ND
25:75	420.3552	a-Tocospiro A	C_29_H_50_O_4_	37780 ± 77	ND
26:38	402.3502	d-Tocopherol	C_27_H_46_O_2_	150789 ± 1745	ND
27:71	430.3811	dl-a-Tocopherol^a^	C_29_H_50_O_2_	8263817 ± 3581	804002 ±1368
29:71	430.3813	Vitamin E	C_29_H_50_O_2_	ND	213437 ± 945

Values are reported as mean ± ± SD;

a Samples differ significantly (*p* < 0.5);

b No significant difference (*p* ≥ 0.5); RT- retention time; ND - not detected

## 2 Experimental Design, Materials and Methods

### 2.1 Sample Preparation

MOLP was obtained from the ARC, Pretoria, South Africa. The MOLP was decolourised by homogenising the leaf powder with ethanol (95%) at a powder to solvent ratio of 1:20, placed on an orbital shaker (Stuart SSL1, Keison Products, Essex, UK) at 150 rpm for 30 min. The samples were later centrifuged, supernatant discarded, and the slurry dried at 40 °C. Further analysis was conducted on both the raw and the decolourised samples

### 2.2 Extraction of Metabolites and GC-HRTOF-MS Analysis

Metabolites were extracted as previously described by Oyedeji, Chinma, Green and Adebo [2]. Ten millilitres of the extraction solvent (methanol/water at 80:20 v/v) together with 1 g each of MOLP samples were thoroughly agitated and sonicated in an ultrasonic bath (Scientech 704, Labotech, South Africa) for 1 h at 4 °C. The mixture was then centrifuged at 3500 rpm at 4 °C for 5 min (Eppendorf 5702R, Merck, South Africa). Supernatant obtained after centrifuging was concentrated in a vacuum concentrator (Eppendorf Plus, Merck, South Africa) and made into solution with 1 ml of chromatographic grade methanol (Merck, South Africa). The solution was vortexed and filtered through 0.22 μm microfilters into an amber vial and solvent blanks were also prepared. The extracts were analysed using a GC-HRTOF-MS (LECO Corporation, St Joseph, MI, USA) with a multipurpose sample (Gerstel Inc., Mülheim an der Ruhr Germany) and Rxi ®-5 ms column (30 m × 0.25 mm ID × 0.25 μm) (Restek, Bellefonte, USA). Injection of 1 µl extract was done a splitless mode at a flowrate of 1 ml/min and helium used as the carrier gas. The ion source temperature was at 250 °C while the transfer line and inlet temperatures were set at 225 and 250 °C, respectively. The oven temperature cycle used was initial temperature of 70 °C for 0.5 min; then an increase of 10 °C/min to 150 °C held for 2 min; then ramped at 10 °C/min to 330 °C and held for 3 min for the column to ‘bake-out’. Data obtained were processed using DataPrep Solutions and metabolites were identified by matching the spectra with NIST, Mainlib and Feihn reference library databases, and their identities determined. Table 1 represents the mean of values obtained from triplicate runs of samples after prior processing of raw data.

## Ethics Statements

None.

## CRediT authorship contribution statement

**Adewumi Toyin Oyeyinka:** Conceptualization, Methodology, Writing – original draft. **Oluwafemi Ayodeji Adebo:** Data curation, Software, Writing – review & editing. **Muthulisi Siwela:** Funding acquisition, Supervision, Writing – review & editing. **Kirthee Pillay:** Funding acquisition, Supervision, Writing – review & editing.

## Declaration of Competing Interest

The authors declare that they have no known competing financial interests or personal relationships that could have appeared to influence the work reported in this paper.

## Data Availability

Supplementary data for manuscript `Dataset on effect of decolourisation on metabolomic profile of Moringa oleifera leaf powder' (Original data) (Mendeley Data)
